# Comparison and Assessment of Different Interatomic Potentials for Simulation of Silicon Carbide

**DOI:** 10.3390/ma17010150

**Published:** 2023-12-27

**Authors:** Jiajie Yu, Xiyue Dai, Jiayuan Li, Anqi Luo, Yifang Ouyang, Yulu Zhou

**Affiliations:** Center on Nanoenergy Research, Guangxi Colleges and Universities Key Laboratory of Blue Energy and Systems Integration, Carbon Peak and Neutrality Science and Technology Development Institute, School of Physical Science & Technology, Guangxi University, Nanning 530004, China

**Keywords:** silicon carbide, interatomic potentials, mechanical properties, thermal properties, defects, molecular dynamics simulation

## Abstract

Interatomic potentials play a crucial role in the molecular dynamics (MD) simulation of silicon carbide (SiC). However, the ability of interatomic potentials to accurately describe certain physical properties of SiC has yet to be confirmed, particularly for hexagonal SiC. In this study, the mechanical, thermal, and defect properties of four SiC structures (3C-, 2H-, 4H-, and 6H-SiC) have been calculated with multiple interatomic potentials using the MD method, and then compared with the results obtained from density functional theory and experiments to assess the descriptive capabilities of these interatomic potentials. The results indicate that the T05 potential is suitable for describing the elastic constant and modulus of SiC. Thermal calculations show that the Vashishta, environment-dependent interatomic potential (EDIP), and modified embedded atom method (MEAM) potentials effectively describe the vibrational properties of SiC, and the T90 potential provides a better description of the thermal conductivity of SiC. The EDIP potential has a significant advantage in describing point defect formation energy in hexagonal SiC, and the GW potential is suitable for describing vacancy migration in hexagonal SiC. Furthermore, the T90 and T94 potentials can effectively predict the surface energies of the three low-index surfaces of 3C-SiC, and the Vashishta potential exhibits excellent capabilities in describing stacking fault properties in SiC. This work will be helpful for selecting a potential for SiC simulations.

## 1. Introduction

Silicon carbide (SiC) possesses excellent electrical properties and corrosion resistance, thereby holding significant industrial value. SiC is often employed as a cladding material in nuclear reactors [1,2,3,4] and widely used in the semiconductor field [5,6,7]. In nature, SiC exists in various crystal structures, including cubic zinc blende (3C) and hexagonal wurtzite (2H, 4H, and 6H). In recent years, first-principles (FPs) calculations of SiC have primarily focused on the electronic band structures, elastic constants, and other properties [8,9,10]. However, because of the high computational cost of FPs calculations, the size of the research system is limited. Consequently, empirical potentials suitable for SiC for large-scale molecular dynamics (MD) simulations have been proposed. Commonly used interatomic potentials include T05 [11], T94 [12], T90 [13], T89 [14], Vashishta [15], environment-dependent interatomic potential (EDIP) [16], modified embedded atom method (MEAM) [17], and GW [18].

These interatomic potentials have different characteristics and applications in simulations. The Tersoff potential accurately describes the covalent bonds of C-Si [19], which are predominant in the bonding character of SiC. Therefore, the Tersoff potentials have been used to simulate mechanical phenomena in SiC, such as tension [20], shear [21], and impact simulations [22]. The Vashishta potential is well-suited for accurately modeling the deformation, both bending and stretching, of ionic and covalent bonds in 3C-SiC [15]. It is extensively used in simulations involving the impact behavior [23] and nanoindentation [24] of SiC. The EDIP potential can accurately describe the transition from covalent to metallic bonding, making it suitable for simulations of amorphous SiC phases [16]. MEAM improves upon the embedded atom method by adding angular-dependent terms to better capture the directional bonding characteristics and has been used in crystal growth [17]. The GW potential, similar in expression format to the Tersoff potential and accounting for stable defect configurations [18], is primarily used in cascade collisions [25].

The parameters of the interatomic potential are determined by fitting to certain physical properties. For instance, Vashishta potential fitted the cohesive energy, bulk modulus, and *C*_11_ elastic constant of 3C-SiC [15], and GW potential has performed fittings for the cohesive energy, lattice constant, and bulk modulus [18]. However, for physical properties not involved in the parameter fitting, the description of these properties by the interatomic potential remains to be validated. Therefore, it is necessary to assess the descriptive ability of the potentials before applying them. The results calculated using the potentials are usually compared with the experimental or density functional theory (DFT) results.

Researchers have predicted and compared some interatomic potentials of properties related to 3C-SiC [11,16,17,26], such as elastic constants and moduli. As determined, T05 provides good results of the elastic constants of 3C-SiC [11,16,27]. Moreover, Vashishta potential shows agreement with experimental results, except for *C*_44_ for 3C-SiC [27]. In the field of thermal properties, most studies have focused on the thermal conductivity of 3C-SiC [28,29,30,31,32,33,34,35] and 4H-SiC [35]. The research revealed that compared with T89 potential, the thermal conductivity predictions by GW potential closely match the experimental results [32]. In addition, studies using interatomic potentials to calculate phonon dispersion in SiC have focused on 3C-SiC [19,35]. The results indicate that Tersoff overestimates the optical branch of the dispersion curve [19,36], and compared with GW, MEAM, T05, and T89 potentials, T90 potential is better in describing phonon thermal transport [35]. However, there were only few comparisons of the prediction with these interatomic potentials on the elastic mechanics, thermal conductivity, and phonon dispersion curves for hexagonal SiC.

Defect properties have a significant impact on the evolution of materials. For example, the formation energies of defects can help assess their stability, and the migration barriers contribute to understanding their migration mechanisms. Some interatomic potentials have been employed to predict and compare the formation energies of point defects in 3C-SiC [11,16,17,18]. The study demonstrated that the GW interatomic potential closely mirrors the ab initio calculations in describing C dumbbell interstitials and antisite defects [18]. The T05 potential tends to underestimate the formation energy of vacancy defects [11], and EDIP potential can better describe C interstitial defects of 3C-SiC [16]. As for point defect migration, compared with the T94 potential, the GW potential is more suitable for predicting carbon atom vacancy–interstitial recombination [37]. For 4H-SiC, empirical potential has been employed to investigate the threshold displacement energies and displacement cascades using MD simulation [38]. However, predictions of properties for defects in hexagonal SiC such as 4H-SiC are rarely mentioned. Regarding planar defects such as generalized stacking fault, Li et al. compared the descriptions of the generalized stacking fault energy (GSFE) using the Vashishta and T05 potentials and concluded that the Vashishta potential is more suitable for describing GSFE in 3C-SiC [27]. Compared to T94, T05 exhibits a closer approximation to the DFT calculation in predicting the unstable GSFE of <112>{111} for 3C-SiC [39]. Xue et al. calculated the GSFE of 4H-SiC using the Vashishta potential and suggested that the easy slip in the <$11\;\bar{2}0$> direction is caused by its significantly low GSFE [24]. The shape of GSFE determines the nature of dislocation cores [40], and thus the descriptions of GSFE in hexagonal SiC by other potentials deserve further explorations.

Motivated by the aforementioned research status, we present predictions of multiple interatomic potentials for the elastic, thermal, and defect properties of four SiC structures with MD methods. These predictions include elastic constants, moduli, Vickers hardness, Debye temperature, vibrational properties, thermal conductivity, point defect formation energies, point defect migration energy barriers, surface energy, and GSFE. In addition, based on DFT and experiments results, a comprehensive comparison of these interatomic potentials in describing the physical properties has been conducted. This evaluation aims to provide insights into the performance of different interatomic potentials in describing properties of SiC. This study provides guidance for the selection of interatomic potentials in SiC simulations.

## 2. Calculation Details

All the calculations with potentials were performed using the Large-scale Atomic/Molecular Massively Parallel Simulator (LAMMPS) software package (version 24Dec2020) [41]. The interactions between C-C, Si-Si, and C-Si were described with various interatomic potentials, namely T05, T94, T90, T89, Vashishta, EDIP, MEAM, and GW. The T05, T94, T90, and T89 potentials are all part of the Tersoff potential family.

### 2.1. Mechanical Properties Calculation

The mechanical characteristics are crucial criteria for assessing the quality of interatomic potential. Among these, elasticity is a vital mechanical property, serving as a criterion for mechanical stability, intrinsic ductility/brittleness, and fracture toughness assessment. To address this, we calculate the elastic constants (*C_ij_*) of SiC. For the models of 3C-, 2H-, 4H-, and 6H-SiC, conventional unit cells were used as repeating units, with expansion performed using the following repeat patterns: 8 × 8 × 8, 8 × 8 × 8, 8 × 8 × 8, and 12 × 12 × 2, respectively. The essential parameters, including the bulk modulus (*B*), shear modulus (*G*), Young’s modulus (*Y*), and Poisson’s ratio (ν), were derived based on the elastic constants. Among these, *B* and *G* were obtained using the Hill average method [42]. The bulk modulus *B*, Young’s modulus *Y*, and shear modulus *G* are obtained using the following formulas [43].

The cubic structure (3C-SiC) is given by

(1) $$B_{V}=B_{R}=(C_{11}+2C_{12})/3$$

(2) $$G_{V}=(C_{11}-C_{12}+3C_{44})/5$$

(3) $$G_{R}=5(C_{11}-C_{12})C_{44}/[4C_{44}+3(C_{11}-C_{12})]$$

(4) $$G_{H}=(G_{V}+G_{R})/2$$

and the hexagonal structure (2H-, 4H-, and 6H-SiC) given by

(5) $$B_{V}=(1/9)[2(C_{11}+C_{12})+4C_{13}+C_{33}]$$

(6) $$G_{R}=(5/2)[C^{2}C_{44}C_{66}]/[3B_{V}C_{44}C_{66}+C^{2}(C_{44}+C_{66})],\;G_{V}=(1/30)(M+12C_{44}+12C_{66})$$

(7) $$B_{R}=C^{2}/M$$

(8) $$G_{H}=(G_{V}+G_{R})/2$$

(9) $$B_{H}=(B_{V}+B_{R})/2$$

(10) $$M=C_{11}+C_{12}+3C_{33}-4C_{13}$$

(11) $$C^{2}=(C_{11}+C_{12})C_{33}-2C_{13}^{2}$$

The Young’s modulus *Y* and the Poisson’s ratio ν are given by

(12) $$Y={\left[1/(3G)+1/(9B)\right]}^{-1}$$

and

(13) ν=(1/2)[1−3G/(3B+G)]

The Vickers hardness can be obtained from elastic properties. Because SiC is a ceramic material, Vickers hardness is calculated using the following formula [44]:

(14) $$H_{V}=2{\left(G^{3}/B^{2}\right)}^{0.585}-3$$

### 2.2. Thermal Properties Calculation

In SiC, transverse velocity (*v_t_*), longitudinal velocity (*v_l_*), and average velocity (*v_m_*) are given by

(15) $$v_{t}={\left(\frac{G}{\rho }\right)}^{\frac{1}{2}},\;v_{l}={\left(\frac{B+\frac{4}{3}G}{\rho }\right)}^{\frac{1}{2}},\;v_{m}={\left[\frac{1}{3}\left(\frac{2}{v_{T}^{3}}+\frac{1}{v_{L}^{3}}\right)\right]}^{-\frac{1}{3}}$$

(16) $$\theta_{D}=\frac{\hbar }{k_{B}}{\left[6\pi^{2}n(\frac{N_{A}\rho }{M})\right]}^{1/3}v_{m}$$

where *ρ* represents the density of the crystal, *n* is the number of atoms in the molecular formula, *N*_A_ is Avogadro’s number, *k*_B_ is the Boltzmann constant, and *M* is the molecular weight.

**Figure 1:**
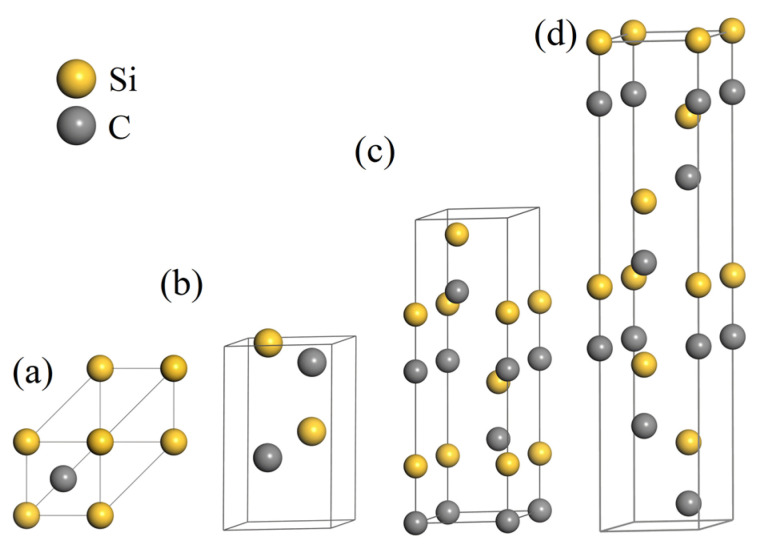
The primitive cell model for (**a**) 3C-SiC; (**b**) 2H-SiC, (**c**) 4H-SiC, and (**d**) 6H-SiC.

For the calculation of phonon dispersion curves, the primitive cell, as shown in Figure 1, was employed as the repeating unit, with repeat patterns of 8 × 8 × 8 (3C), 8 × 8 × 8 (2H), 8 × 8 × 8 (4H), and 12 × 12 × 2 (6H), following the method proposed by Kong et al. [45]. Periodic boundary conditions were applied in all three directions. The time step was set to 0.0002 ps. The microcanonical ensemble (NVE) was chosen, and the fix phonon [45] command was applied to the entire system during the calculation process. A time step of 0.002 ps was used, and the measurement of Green’s function was conducted every 10 MD steps in the following 12,000 ps. After obtaining the dynamic matrix, PHANA [45] and PHONOPY [46] codes were used to calculate the phonon frequency.

The phonon dispersion curves were also calculated by DFT with the VASP package (version 6.1.0) [47]. The generalized gradient approximation (GGA) in the form of Perdew–Burke–Enzerhof (PBE) [48] exchange-correlation energy functional was used, and the cutoff energy was set to 600 eV. The energy convergence standard was set at 1.0 × 10^−8^ eV. The model was constructed based on protocells, as the repeating unit, and employed expansion patterns of 3 × 3 × 3 (3C), 3 × 3 × 2 (2H), 3 × 3 × 1 (4H), and 3 × 3 × 1 (6H). The 5 × 5 × 5 *k*-meshes were used in the Brillouin region.

The molar heat capacity *C_V_* and entropy *S* can be calculated by PHONOPY, and the calculation formula is as follows [49]:

(17) $$C_{V}=\sum_{qj}C_{qj}=\sum_{qj}k_{B}{\left(\frac{\hbar \omega_{qj}}{k_{B}T}\right)}^{2}\frac{\exp(\frac{\hbar \omega_{qj}}{k_{B}T})}{{\left[\exp(\frac{\hbar \omega_{qj}}{k_{B}T})-1\right]}^{2}}$$

(18) $$S=\frac{1}{2T}\sum_{qj}\hbar \omega_{qj}\coth\left[\hbar \omega_{qj}/2k_{B}T\right]-k_{B}\sum_{qj}\ln\left[2\sinh\left(\hbar \omega_{qj}/2k_{B}T\right)\right]$$

where $\omega_{qj}$ is the frequency calculated by $D(q){\vec{e}}_{qj}=\omega_{qj}^{2}{\vec{e}}_{qj}$ and *D* is the dynamical matrix.

Thermal conductivities were calculated using equilibrium molecular dynamics (EMD) based on Green’s function approach. The expression for thermal conductivity calculation can be derived from the Green’s function as follows:

(19) $$\kappa =\frac{V}{k_{B}T^{2}}\int_{0}^{\infty }<J(0)J(t)>dt$$

where *k*_B_, *T*, *V*, and <J(0)J(t)> represent the Boltzmann constant, temperature, volume of the model, and the heat flux autocorrelation function, respectively.

For 3C-SiC, thermal conductivities were averaged across the [100], [010], and [001] directions, while for hexagonal structures, thermal conductivities were calculated separately for both in-plane and out-of-plane directions. In the thermal conductivity calculations, the number of atoms in the models for 3C-SiC, 2H-SiC, 4H-SiC, and 6H-SiC were 1728, 1728, 2400, and 2400, respectively. Convergence can typically be achieved to approximately 1500 atoms when calculating thermal conductivity using the EMD method [50]. During the calculations, we used a time step of 0.0002 ps. After relaxing for 106 steps (200 ps) in the NPT ensemble, followed by an additional 106 steps at least in the NVE ensemble, data collection was performed in the NVE ensemble. The results are the average of five independent simulations. Owing to the difficulty in achieving convergence for the heat flux autocorrelation function at 300 K, data collection was performed for 12,000 ps when the temperature was at T = 300 K. For temperatures T > 300 K, data collection was performed for 6000 ps.

### 2.3. Defect Properties Calculation

This study primarily focuses on the formation energies of point defects in SiC, including V_C_ (C vacancy), V_Si_ (Si vacancy), substitutional defect C_Si_ (C occupying Si site), substitutional defect Si_C_ (Si occupying C site), SiT_C_ (Si occupying C tetrahedral site), CT_Si_ (C occupying Si tetrahedral site), and eight types of dumbbell interstitials. The conjugate gradient (cg) method was used to minimize the energy. The timestep was set to 0.001 ps. The formation energies of a single vacancy $E_{f}^{V_{A}}$, interstitial defect $E_{f}^{I_{A}}$, and substitutional defect $E_{f}^{A_{B}}$ were given as follows:

(20) $$E_{f}^{V_{A}}=E_{d}-E_{\mathrm{perfect}}+E(A)$$

(21) $$E_{f}^{I_{A}}=E_{d}-E_{\mathrm{perfect}}-E(A)$$

(22) $$E_{f}^{{}_{A_{B}}}=E_{d}-E_{\mathrm{perfect}}-E(A)+E(B)$$

where A and B represent C or Si, respectively, and *E*(A) and *E*(B) are the energy per atom in the bulk phase (diamond structures of C and Si). *E*_d_ and *E*_perfect_ represent the energy of the system with defects and without defects, respectively.

The migration energies of point defects were estimated by means of the nudged elastic band (NEB) method with ‘quickmin’ minimization style. The timestep was set to 0.0001 ps. The model of 3C-SiC consists of 8 × 8 × 8 unit cells along X[100], Y[010], and Z[001] directions. The models of hexagonal SiC consist of 8 × 8 × 8 (2H), 8 × 8 × 8 (4H), and 10 × 10 × 5 (6H) unit cells along X$\left[2\bar{1}\bar{1}0\right]$, Y$\left[\bar{1}2\bar{1}0\right]$, and Z[0001] directions, respectively.

The calculation of GSFE refers to the method of Vitek et al. [51]. The model of 3C-SiC consists of 10 × 10 × 20 unit cells along X$\left[1\bar{1}0\right]$, Y$\left[11\bar{2}\right]$, and Z[111] directions, respectively. The models of hexagonal SiC consist of 10 × 10 × 20 (2H), 10 × 10 × 10 (4H), and 10 × 10 × 6 (6H) unit cells along X$\left[2\bar{1}\bar{1}0\right]$, Y$\left[01\bar{1}0\right]$, and Z[0001] directions, respectively. The GSFE of 3C-SiC were calculated for displacements along the $\left[1\bar{1}0\right]$ and $\left[11\bar{2}\right]$ directions in the (111) plane, and hexagonal SiC were calculated for displacements along the $\left[2\bar{1}\bar{1}0\right]$ direction in the (0001) plane. The timestep was set to 0.005 ps.

To provide reference results for the FPs calculations of point defect formation energy, defect migration energy, and GSFE of hexagonal SiC, we employed the VASP software with using the GGA in the PBE parameterization. For the calculation of point defect formation energy, the energy cutoff was set to 500 eV [52]. When calculating defect migration energy and GSFE, the energy cutoff was set to 600 eV. The force convergence criterion was set to 0.01 eV/Å. The method of climbing image nudge elastic band (CI-NEB) was used to calculate the defect migration energy [53].

## 3. Results and Discussion

### 3.1. Mechanical Properties

Before the examination of mechanical properties, a comprehensive understanding of the fundamental structural attributes of SiC under the influence of diverse interatomic potentials was established. Lattice constants and cohesive energies for the four SiC structures were computed, and the results are presented in Table 1. The cubic phase (3C-SiC) and the hexagonal phases (2H-, 4H-, and 6H-SiC) exhibit similar cohesive energy (*E*_c_) values. Except for T94, other interatomic potentials describe the lattice constants of SiC well.

**Figure 2:**
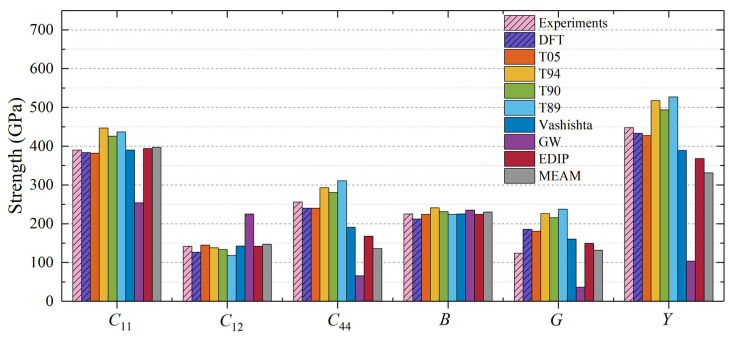
Elastic constants and moduli for 3C-SiC calculated using different interatomic potentials.

The calculated bulk modulus *B* values for 3C-SiC, which closely match the DFT and experimental results, are illustrated in Figure 2. This is because most interatomic potentials are constructed using Young’s modulus as a fitting parameter. The elastic constants of all interatomic potentials satisfy the following mechanical stability criteria [43]: *C*_11_ > 0, *C*_11_ − *C*_12_ > 0, *C*_44_ > 0, *C*_12_ + 2*C*_12_ > 0. The T05 potential exhibits excellent descriptive capabilities, showing strong consistency with the DFT results. In comparison, T89, T90, and T94 potential slightly overestimate *C*_44_ and the shear modulus *G*. The GW potential does not perform as well as Tersoff potentials regarding describing elastic constants and moduli. This difference may be attributed to the fact that GW potential was developed primarily to describe the formation energy of point defects [16,25] rather than mechanical properties. The calculated values of *C*_11_ and *C*_12_ obtained through Vashishta, EDIP, and MEAM potentials show good agreement with experimental results. However, Vashishta, EDIP, and MEAM potentials underestimate *C*_44_, which consequently leads to a lower Young’s modulus *Y*.

Although 2H-SiC consists of a 100% hexagonal structure, which is fundamentally different from the 100% cubic structure of 3C-SiC, the calculated bulk modulus *B* for 2H-SiC closely agrees with the experimental and DFT results. This implies that the description of the bulk modulus *B* by SiC interatomic potentials remains largely unaffected by the differences in crystal structures. For 2H-SiC, the mechanical properties calculated using the Tersoff potential show excellent agreement with the experimental and DFT results. In addition, the MEAM potential tends to overestimate *C*_12_ and *C*_33_ while significantly underestimating *C*_13_ for 2H-SiC.

**Figure 3:**
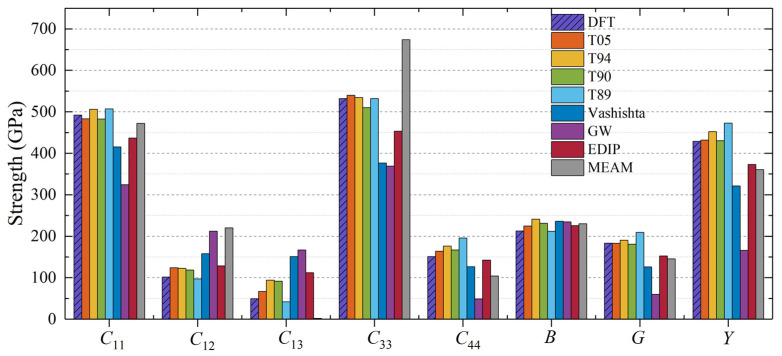
Elastic constants and moduli for 2H-SiC calculated using different interatomic potentials.

**Figure 4:**
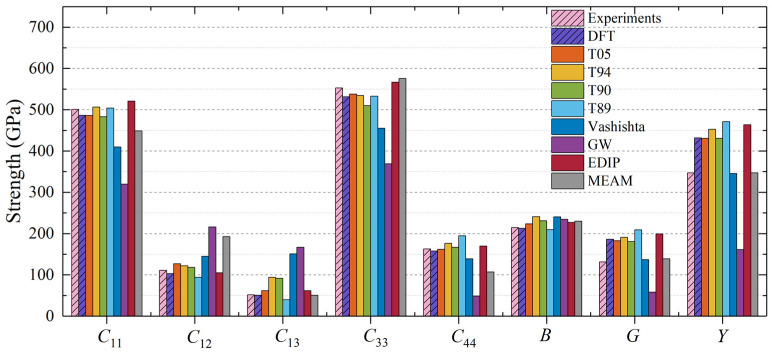
Elastic constants and moduli for 4H-SiC calculated using different interatomic potentials.

**Figure 5:**
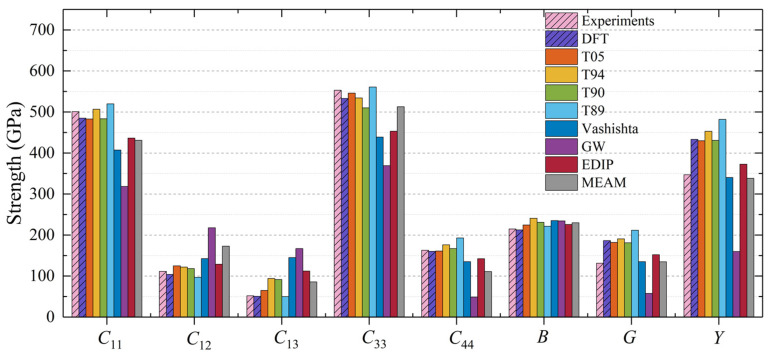
Elastic constants and moduli for 6H-SiC calculated using different interatomic potentials.

In the case of 4H-SiC, the moduli are in excellent agreement with the experimental values. Comparing the results for the three hexagonal phases (Figure 3, Figure 4 and Figure 5), except for the MEAM and EDIP potentials, the elastic properties of 4H-SiC, 6H-SiC, and 2H-SiC are quite similar under the interactions of the other interatomic potentials, aligning well with the DFT results. By comparing the elastic constants of 2H-SiC, 4H-SiC (50% hexagonality), and 6H-SiC (33% hexagonality) obtained using the MEAM and EDIP potentials, it can be observed that, unlike other interatomic potentials, the elastic constants of these three phases are different. This indicates that the mechanical properties are influenced by the different structures when using these two interatomic potentials.

To conduct a more in-depth assessment of these interatomic potentials, we calculated Poisson’s ratio, *B*/*G* ratio, and Vickers hardness based on the elastic constants. Poisson’s ratio can be used to predict the shear stability of materials and distinguish whether a material is brittle or ductile, with 0.33 as the boundary point [8]. Table 2 shows that the Poisson’s ratio obtained from the Tersoff, Vashishta, EDIP, and MEAM potentials indicates that 3C-SiC is a brittle material, and the same result holds for other phases (2H, 4H, and 6H) listed in Table 3, Table 4 and Table 5. The value of *B*/*G* can also characterize the toughness of materials, with 1.75 as the boundary to distinguish between brittle and ductile materials. Materials with a *B*/*G* ratio below 1.75 are considered brittle, whereas those above 1.75 are considered ductile [8]. The *B*/*G* ratio of 3C-SiC is also listed in Table 2. Tersoff, Vashishta, and EDIP consistently suggest that 3C-SiC exhibits brittle behavior, which is consistent with the experimental findings. For 3C-SiC, the experimental values for Vickers hardness fall within the range of 26 ± 2 GPa [59]. Tersoff and Vashishta potentials can reasonably predict the Vickers hardness of 3C-SiC, particularly for T05. However, for 2H-, 4H-, and 6H-SiC, Vashishta, GW, and MEAM potentials tend to underestimate the Vickers hardness.

Overall, in terms of the mechanical properties of SiC, including elastic characteristics, brittleness, and hardness, Tersoff potentials provide the most accurate descriptions, whether for the cubic or hexagonal phase. The Vashishta potential closely follows as a strong performer. Although the Vashishta potential relies solely on fitted data referencing cohesive energy, bulk modulus, and the *C*_11_ elastic constant from experimental data [15], it is still suitable for describing the mechanical properties of hexagonal SiC.

### 3.2. Thermal Properties

#### 3.2.1. Debye Temperature

As we know, the Debye temperature is a crucial thermal property of solid materials. It not only provides fundamental characteristics of vibrational spectra but also characterizes properties such as the coefficient of thermal expansion. The calculated sound velocities and Debye temperatures are presented in Table 6. Except for the slightly larger values obtained with T89, all Tersoff potentials, whether in terms of sound velocities or Debye temperatures, are close to the DFT results. Other interatomic potentials generally exhibit slightly smaller values, particularly for the GW potential.

#### 3.2.2. Phonon Dispersion Curve

**Figure 6:**
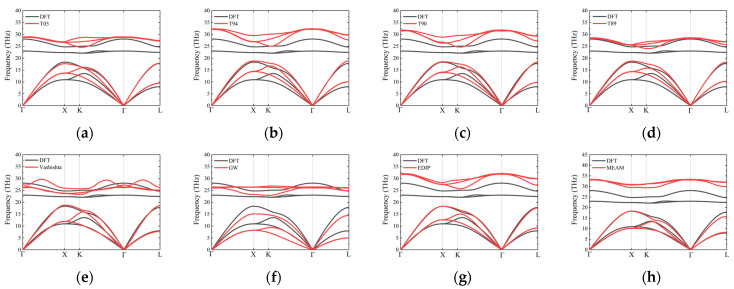
Phonon dispersion along high-symmetry directions for 3C-SiC, obtained using several different potentials: (**a**) T05; (**b**) T94; (**c**) T90; (**d**) T89; (**e**) Vashishta; (**f**) GW; (**g**) EDIP and (**h**) MEAM. Black curves and red curves represent the results of DFT and empirical potentials, respectively.

Phonon dispersion provides crucial insights into the structural and thermal properties of materials. For 3C-SiC, the primary path used is Γ–X–K–Γ–L [36], as show in Figure 6. Overall, none of the interatomic potentials show imaginary frequencies when describing phonon dispersion curves. Some of the outcomes of interatomic potentials exhibit slight variations compared with previous research results [35], which are likely attributed to differences in the selected model sizes. To further investigate the accuracy of the dispersion curves, Table 7 summarizes the phonon frequencies at high-symmetry points for 3C-SiC. Tersoff potentials, namely T05, T94, T90, and T89, provide good descriptions of the longitudinal acoustic (LA) branch. For example, the frequencies of the LA branch at the L point for T05, T94, T90, and T89 are 16.65 THz, 16.65 THz, 17.99 THz, and 17.61 THz, respectively, which agree well with DFT (16.65 THz) and the experimental results (16.95 THz). However, Tersoff potentials do not provide a very accurate description of the transverse acoustic (TA) branch. The frequency of the TA branch at the X point is approximately 27% higher than the experimental results. In addition, the optical branches of their phonon dispersion curves are not well-described. This is because some interatomic potentials, such as Tersoff potentials, only consider the interaction between covalent bonds when proposed. However, SiC also contains 12% ionic bonds; thus, interatomic potentials like Tersoff are unable to accurately describe the optical branches, and the TA branch tends to be overestimated [19]. Previous research found that T90 potential can describe the acoustic branch of phonons well [35], but the acoustic branches obtained using Vashishta, EDIP, and MEAM potentials agree well with the DFT curves. Among them, the frequencies of the optical branches from Vashishta potential are in the same frequency range as those from DFT. This is because both covalent interactions and ionic interactions are taken into account when constructing the Vashishta potentials [15]. This contributes to a relatively accurate calculation of phonon dispersion using the Vashishta potential.

**Figure 7:**
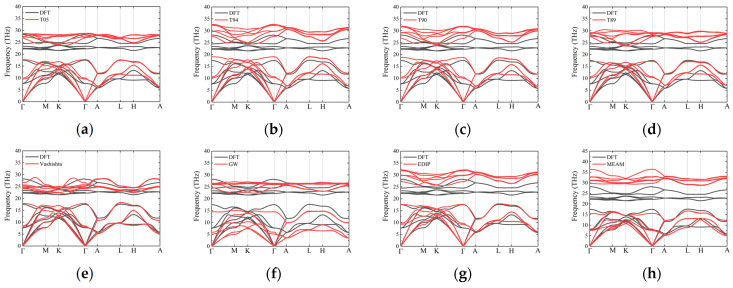
Phonon dispersion along high-symmetry directions for 2H-SiC, obtained using several different potentials: (**a**) T05; (**b**) T94; (**c**) T90; (**d**) T89; (**e**) Vashishta; (**f**) GW; (**g**) EDIP and (**h**) MEAM. Black curves and red curves represent the results of DFT and empirical potentials, respectively.

**Figure 8:**
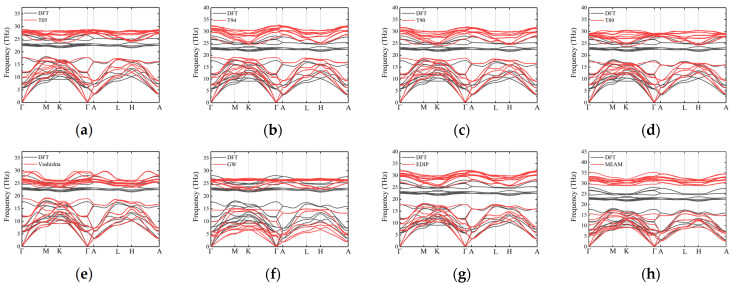
Phonon dispersion along high-symmetry directions for 4H-SiC, obtained using several different potentials: (**a**) T05; (**b**) T94; (**c**) T90; (**d**) T89; (**e**) Vashishta; (**f**) GW; (**g**) EDIP and (**h**) MEAM. Black curves and red curves represent the results of DFT and empirical potentials, respectively.

**Figure 9:**
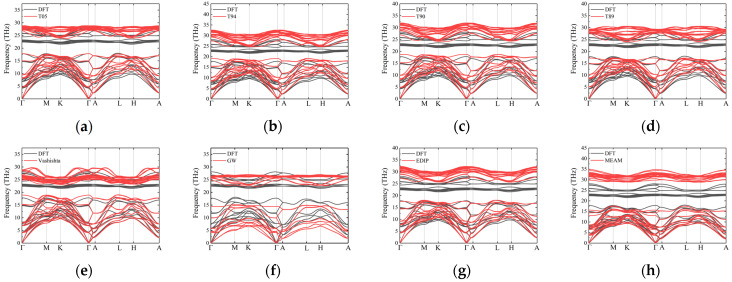
Phonon dispersion along high-symmetry directions for 6H-SiC, obtained using several different potentials: (**a**) T05; (**b**) T94; (**c**) T90; (**d**) T89; (**e**) Vashishta; (**f**) GW; (**g**) EDIP and (**h**) MEAM. Black curves and red curves represent the results of DFT and empirical potentials, respectively.

The phonon dispersion curves for 2H-SiC are shown in Figure 7. Similar to the case of 3C-SiC, none of the interatomic potentials can accurately describe the optical branches. The Tersoff potential overestimates the frequencies of the optical branches and provides a relatively good description of the acoustic branches, particularly the LA branches. For the TA branches, particularly along the L–H path, the frequencies are approximately 18% higher than the DFT curves. GW fails to describe the acoustic branch frequencies of 2H-SiC, and the frequencies at various high-symmetry points are generally lower. The results from the Vashishta potential agree well with the DFT results, particularly for the acoustic branches, and the optical branches also fall within the same frequency range as the DFT optical branches. The results from the EDIP and MEAM potentials are similar, with a good description of the acoustic branches but overestimated frequencies for the optical branches. The results of other hexagonal phases (4H and 6H) are similar to those of 2H-SiC, as shown in Figure 8 and Figure 9.

#### 3.2.3. Heat Capacity and Entropy

**Figure 10:**
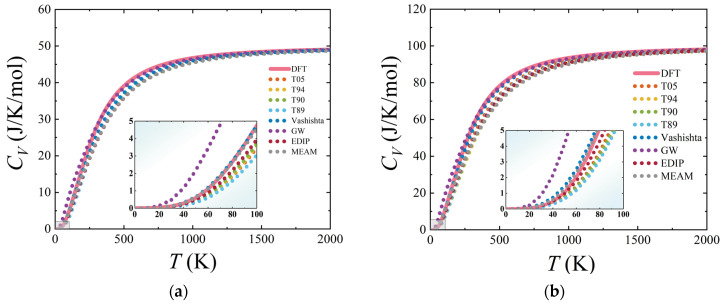

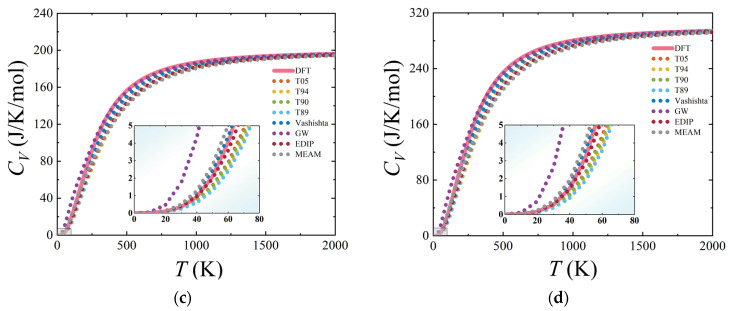
Temperature dependence for entropy *C_V_*. (**a**), (**b**), (**c**), and (**d**) represent the results for 3C-, 2H-, 4H-, and 6H-SiC, respectively. The pink curves represent the results obtained from DFT calculations.

The behavior of the molar heat capacity *C_V_* of 3C-SiC with temperature is depicted in Figure 10a. The trends of the curves for various interatomic potentials are relatively consistent with the DFT results. Each curve exhibits a rapid increase as the temperature rises, and once the temperature surpasses the Debye temperature, they gradually converge to the same value (Dulong–Petit limit). To better illustrate the curve changes, we have zoomed in on the low-temperature region. For 3C-SiC, in the low-temperature range (*T* < 100 K), Vashishta, EDIP, and MEAM potentials align closely with the heat capacity curve derived from DFT calculations. This phenomenon may be attributed to the fact that at low temperatures, only the low-frequency acoustic branches are excited, which means the heat capacity is primarily influenced by low-frequency vibrations. Combining Figure 6e,g,h, it can be inferred that the acoustic branches of the Vashishta, EDIP, and MEAM potentials match well with the DFT results, resulting in a good agreement in their *C_V_* curves with DFT. The acoustic branch frequencies of Tersoff potentials are generally higher than those from DFT. The acoustic branch frequencies of GW potential are lower than the results of DFT, leading to discrepancies in the heat capacity calculated using this potential compared with DFT results. However, at *T* > 300 K, the GW results exhibit a better agreement with DFT results.

Similarly, the heat capacity *C_V_* of hexagonal SiC increases with increasing temperatures, as illustrated in Figure 10b–d. Different SiC structures converge to distinct limits in the high-temperature region. When the temperature *T* < 100 K (low-temperature range), the results from the Vashishta, EDIP, and MEAM potentials are relatively close to the heat capacity curve obtained from DFT calculations. When *T* > 300 K, the results from the GW potential gradually approach the molar heat capacity curve from DFT.

**Figure 11:**
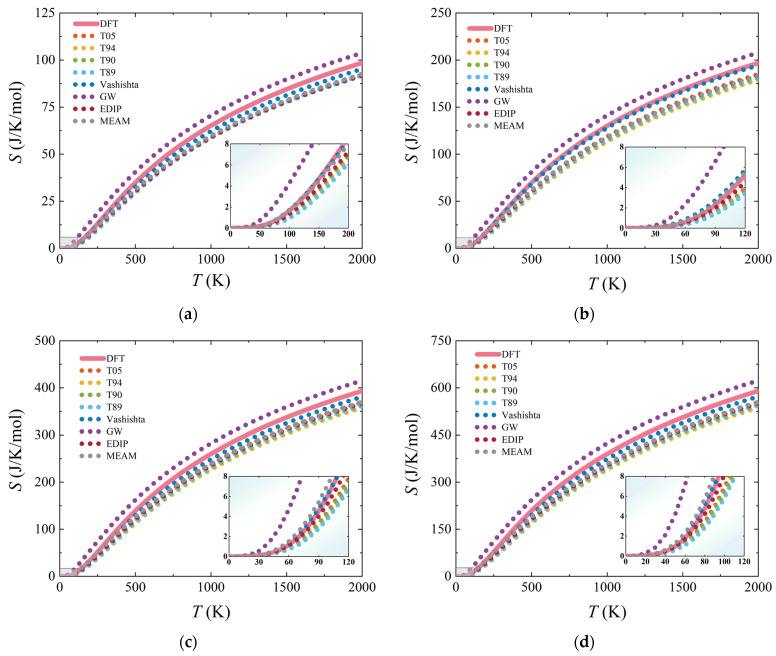
Temperature dependence for entropy *S*. (**a**), (**b**), (**c**), and (**d**) represent the results for 3C-, 2H-, 4H-, and 6H-SiC, respectively. The pink curves represent the results obtained from DFT calculations.

Furthermore, we also calculated the vibrational entropy (*S*) as a function of temperature, and the results are shown in Figure 11. For four SiC structures, all potentials exhibit consistent trends. In the low-temperature region of the *S*–*T* curves, the results of Vashishta, EDIP, and MEAM potentials closely match the DFT calculation. In particular, the Vashishta potential, when describing *S* for 2H-SiC, still closely overlaps with the reference curve even in the high-temperature region.

#### 3.2.4. Thermal Conductivity

**Figure 12:**
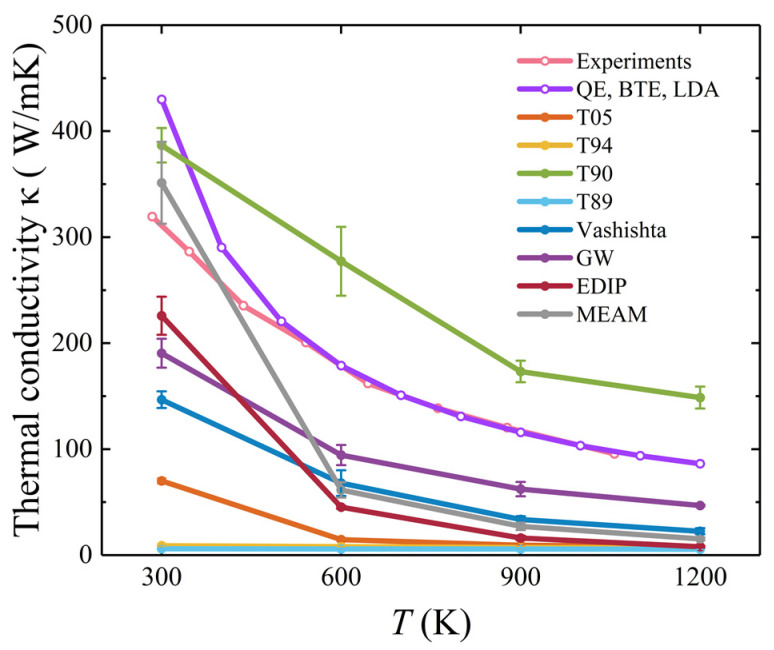
Thermal conductivity for 3C-SiC. The pink and purple lines represent the results of the experiment [68] and DFT [66].

The temperature dependence of the thermal conductivity of 3C-SiC is shown in Figure 12. The predictions of the interatomic potentials, except for the T89 and T94 potentials, decrease significantly with increasing temperature, which is in agreement with the results of previous studies [34,64]. The decrease in thermal conductivity with increasing temperature is attributed to the reduction in the average phonon mean free path. The thermal conductivity obtained with GW and T89 potentials is in agreement with previous studies [32]. In addition, the thermal conductivity calculated using T94 potential closely matches that obtained by previous researchers who employed T94 potential with a Ziegler–Biersack–Littmark short-range correction [34,65]. The results from the MEAM and T90 potentials fall between the DFT [66] and experimental values [67]. When the temperature exceeds 300 K, the results from all interatomic potentials deviate from the DFT and experimental results. Overall, in comparison, T90 and GW potentials show better agreement with the reference values. T94 and T89 potentials tend to significantly underestimate the thermal conductivity of SiC.

**Figure 13:**
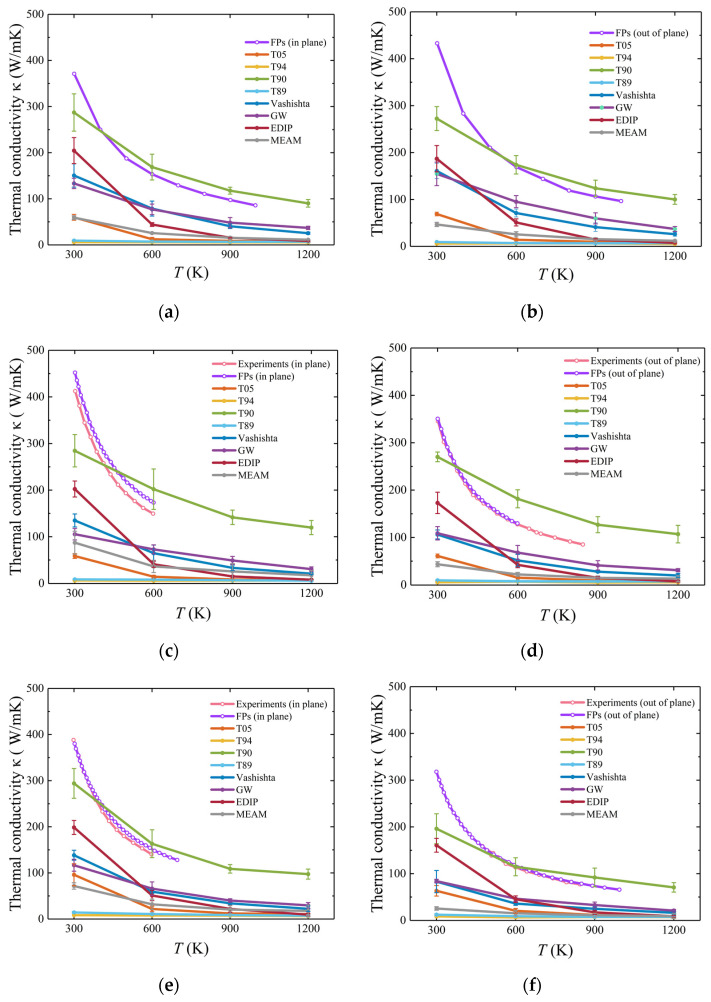
Temperature-dependent thermal conductivity for 2H-, 4H-, and 6H-SiC. The in-plane and out-of-plane thermal conductivities of 2H-SiC are shown in (**a**) and (**b**), respectively. The in-plane and out-of-plane thermal conductivities of 4H-SiC are shown in (**c**) and (**d**), respectively. The in-plane and out-of-plane thermal conductivity of 6H-SiC are shown in (**e**) and (**f**), respectively. The experimental and FPs results are from a previous study [69].

For hexagonal SiC, thermal conductivities in both in-plane and out-of-plane directions were calculated separately, as shown in Figure 13. In comparison, thermal conductivity obtained using T90 potential appears to be closer to the FPs results for the two directions. However, other interatomic potentials tend to significantly underestimate the thermal conductivity of hexagonal SiC, whether in the in-plane or out-of-plane direction.

### 3.3. Defect Properties

#### 3.3.1. Point Defect Formation Energy

The results of the formation energy of defects are presented in Table 8. Because of structural instability observed during DFT calculations for Si-C + <110> (the symbol “+” represents the interstitial atom), data on this structure are not listed in Table 8. In addition, when applying the Vashishta potential to compute the cohesive energy of diamond C phase and diamond Si phase, both C and Si exhibited excessively low cohesive energies, approaching zero, making it impossible to calculate the formation energy of point defects in SiC. Thus, we have opted not to include the defect data associated with the Vashishta potential. To describe vacancies, T94, T90, and GW potentials seem to have an advantage. The GW potential is better suited for describing substitutional defects. It should be noted that when the GW potential was proposed, other formulas were employed to calculate the formation energy of point defects. To ensure a meaningful comparison, we used Equations (20)–(22) to recalculate the formation energy. The GW potential does not obtain a good interstitial defect formation energy because the GW potential cannot describe the energies of pure silicon and pure carbon. Regarding interstitial defects located in tetrahedral sites, only T05 can achieve the best results for CT_Si_ and CT_C_. In the case of dumbbell interstitial defects, T89, T90, and MEAM potentials can describe C-C + <100> well. T05 and T89 potentials can predict the formation energy of Si-C + <100>. Furthermore, T90 and GW potentials provide accurate formation energies for Si-Si + <100> and C-Si + <100>. Overall, it appears that none of these interatomic potentials can effectively predict the formation energies of all dumbbell interstitials. However, based on the description of more easily generated point defects, T94 should be the optimal choice. Compared with other interaction potentials, the formation energies of point defects obtained using GW potential are generally lower. This leads to a higher number of point defects in cascade collisions obtained using the GW potential than those obtained with the T94 potential [37].

**Figure 14:**
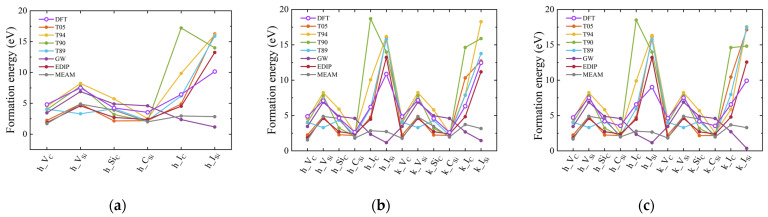
Defect formation energies for different point defects. (**a**–**c**) represent the results for 2H-, 4H-, and 6H-SiC, respectively. The DFT results for 4H-SiC are referenced from a previous study [72]. The h represents the hexagonal environment, and k represents the cubic environment.

The results of formation energies for point defects in hexagonal SiC are presented in Figure 14 and also listed in Tables S1–S3. The structural reference for interstitial defects is taken from a previous study [72]. The DFT results show that the difference in structure has no significant effect on the point defect formation energy in 2H and 6H-SiC. Each has its advantages in describing these defects. The T90, T94, and GW potentials perform well in describing V_C_, V_Si_, and Si_C_. The MEAM and T89 potentials provide formation energies for Si_C_ that are in excellent agreement with DFT results. However, from the perspective of the energy trends, only the results obtained from the T05 and EDIP potentials align with those from DFT. Among these, EDIP potential appears to provide a better description of the defect formation energies in hexagonal SiC.

#### 3.3.2. Point Defect Migration Barrier

The nudged elastic band (NEB) method helps in exploring the diffusion mechanisms of vacancies and interstitials [73]. Through the evaluation of migration pathways and energy barriers associated with point defects, including vacancies and interstitials, the precision of interatomic potentials in characterizing atomic mobility and energy variations can be corroborated.

**Figure 15:**
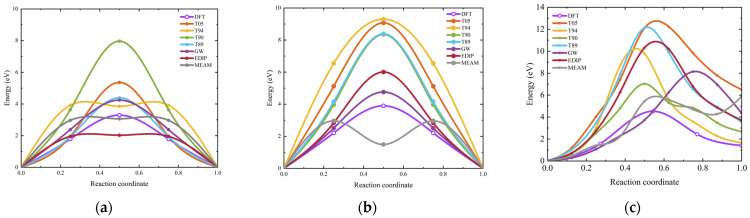
Vacancy migration barrier for 3C-SiC. (**a**–**c**) depict the migration energy barriers for V_C_ → V_C_, V_Si_ → V_Si_, and V_C_–C_Si_ → V_Si_ in 3C-SiC, respectively.

Here, we calculate the migration of vacancies in 3C-SiC along three paths, namely V_C_ → V_C_, V_Si_ → V_Si_, and V_C_–C_Si_ → V_Si_, respectively. The DFT results are also calculated, as shown in Figure 15. The migration barriers for these three paths obtained from DFT calculations are 3.64 eV, 3.51 eV, and 2.41 eV, respectively, which is consistent with the values reported by Bockstedte et al. [74]. For interatomic potentials, in the V_C_ → V_C_ pathway, only the GW potential can obtain relatively good results, followed by the T89 potential. In the V_Si_ → V_Si_ pathway, GW and EDIP potentials yield relatively good results for migration barriers.

**Figure 16:**
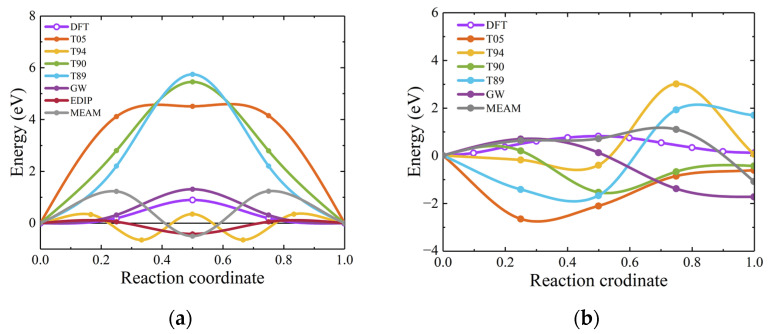
Interstitial migration energy barrier for 3C-SiC. (**a**,**b**) depict the migration of I_C_ along the C_sp_ <100> → C_sp_ <100> path and I_Si_ along the Si_sp_ <110> → Si_TC_ path, respectively, with the DFT results in (**b**) referenced from a previous study [76]. The “sp” represents split interstitial.

Interstitial migration outcomes are illustrated in Figure 16, with (a) and (b) denoting the migration for carbon interstitial (I_C_) and silicon interstitial (I_Si_), respectively. For I_C_, the most favorable migration pathway is C_sp_ <100> → C_sp_ <100> [75]. For I_Si_, the two most favorable interstitial sites are the carbon tetrahedral site and the <110> dumbbell interstitial [74]. In addition, Si_sp_ <110> → Si_TC_ is an essential constituent unit for the long-range migration of neutral silicon interstitial [76]. We calculated the energy barriers for both C_sp_ <100> → C_sp_ <100> and Si_sp_ <110> → Si_TC_ migration pathways. In the C_sp_ <100> → C_sp_ <100> pathway, the energy barrier calculated through DFT is found to be 0.89 eV, which is very close to the value of 0.88 eV in a previous study [75]. The results show that the GW potential describes the migration of I_C_ well, particularly in terms of the barrier height. The results for I_Si_ migration from Si_sp_ <110> to Si_TC_ also show that the empirical potentials differ significantly from the DFT calculations, particularly in the description of the saddle point barrier. During migration, some of these interatomic potentials yield negative values, indicating that the structures of the initial and final states of migration are in metastable states.

**Figure 17:**
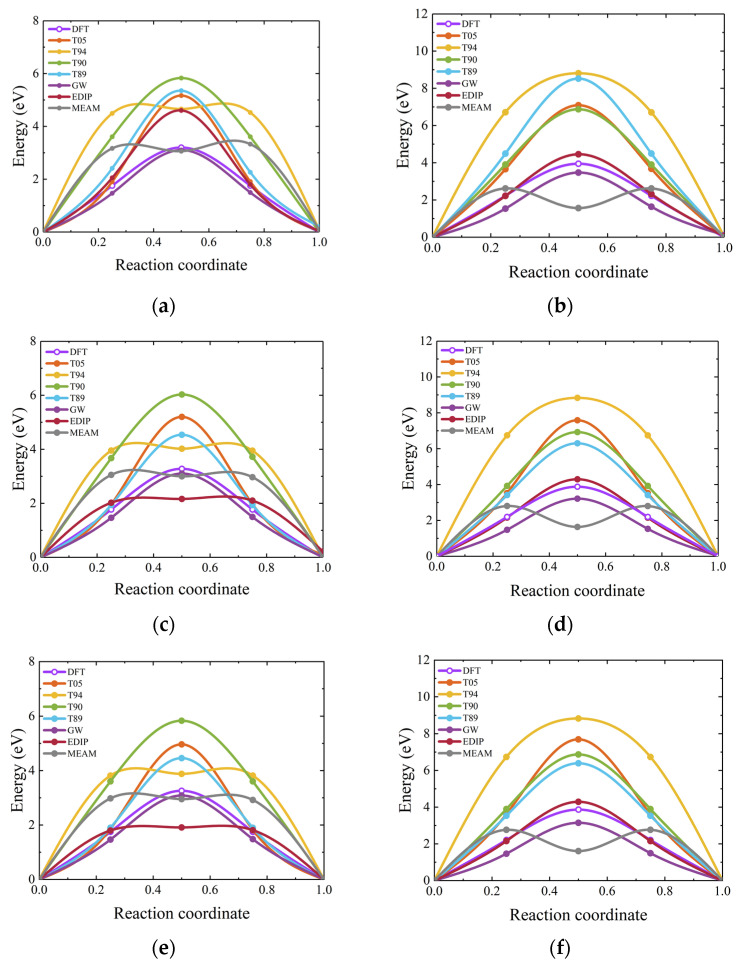
Vacancy migration barrier for hexagonal SiC. (**a**,**b**), (**c**,**d**), and (**e**,**f**) illustrate the migration energy barriers for V_C_ → V_C_ and V_Si_ → V_Si_ in 2H-, 4H-, and 6H-SiC, respectively.

In 2H-, 4H-, and 6H-SiC, we primarily investigate the V_C_ and V_Si_ in a hexagonal structural environment. The DFT reference results for 2H-, 4H-, and 6H-SiC are calculated by the current study. For the migration barriers of V_C_ and V_Si_ in 4H-SiC, the DFT results are 3.30 eV and 3.90 eV, respectively, consistent with the values obtained in previous research using the GGA and HSE hybrid functional, which were 3.31 eV for V_C_ and 4.32 eV for V_Si_ [77]. The results in Figure 17 indicate that the empirical potential exhibits good symmetry for V_C_ migration, but only GW potential provides accurate results for the energy barrier calculation in hexagonal SiC. When describing the migration behavior of V_Si_ in hexagonal SiC, both the GW and EDIP potentials demonstrate excellent descriptive capabilities. The main features are that the migration path exhibits good symmetry, and the energy barrier of the saddle point matches the DFT value. In contrast, other interatomic potentials tend to overestimate or underestimate the migration energy barrier. Overall, GW potential is well-suited for describing vacancy migration in hexagonal SiC.

#### 3.3.3. Surface Energy and GSFE

**Figure 18:**
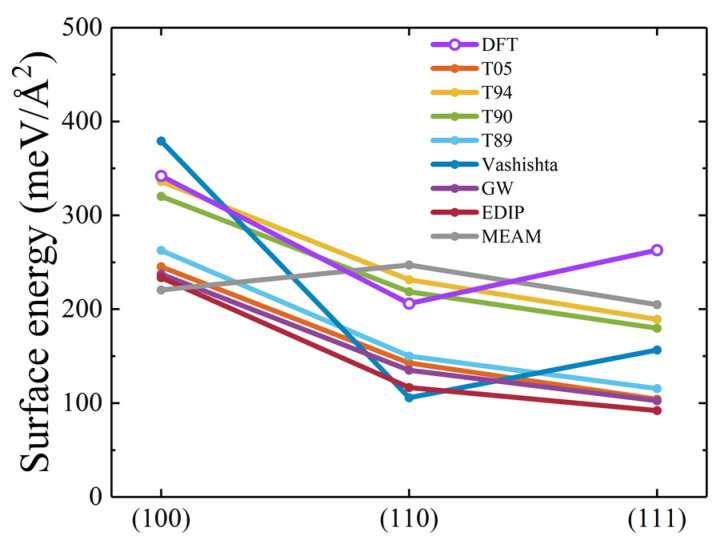
Surface energies of three low-index surfaces for 3C-SiC. The DFT, T05, and Vashishta data are taken from a previous study [27].

Surface is also a very important defect in materials [78]. Furthermore, in nanoscale indentation and similar processes, surface energy can also serve as a measure of deformation on low-index crystallographic planes [27]. The calculated surface energy results for 3C-SiC are shown in Figure 18. It can be seen that the surface energies of (100) and (110) surfaces calculated by T94 and T90 potentials are in excellent agreement with DFT results. In the case of the (111) surface, the result obtained through the MEAM potential is the closest to DFT, followed by those from T90 and T94 potentials. Overall, T90 and T94 potentials exhibit good capabilities in describing the formation energies of the three low-index surfaces in 3C-SiC.

**Figure 19:**
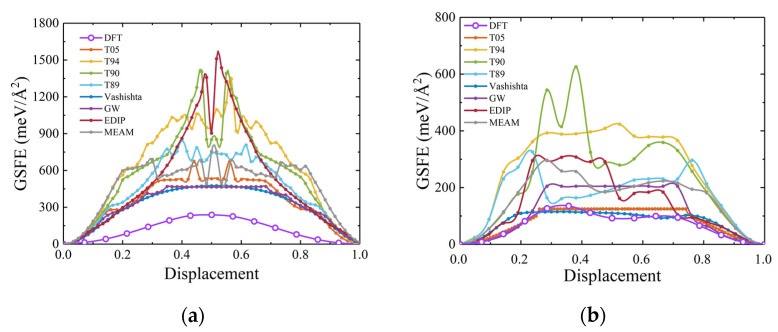
Generalized stacking fault energy for 3C-SiC. (**a**,**b**) show the GSF energies for movement along the $\left[1\bar{1}0\right]$ and $\left[11\bar{2}\right]$ directions on the (111) plane, with data from DFT, T05, and Vashishta taken from a previous study [27].

The GSFE is one of planar defects that control fracture behavior and determine dislocation properties [51,78]. Furthermore, it represents the energy required for lattice movement along a specific plane and can serve as an indicator for evaluating dislocation slip [24]. This study evaluates these interatomic potentials for stacking fault energy by calculating the GSFE of SiC. Considering the slip systems of 3C-SiC, calculations were performed on the (111) plane along both the $\left[1\bar{1}0\right]$ and $\left[11\bar{2}\right]$ directions. The results in Figure 19 show that the displacements are normalized. It can be observed that the empirical potentials tend to overestimate the GSFE in the $\left[1\bar{1}0\right]$ direction. In the $\left[11\bar{2}\right]$ direction, the T05 and Vashishta potentials exhibit energy values consistent with those obtained from DFT, with Vashishta potential being notably consistent.

**Figure 20:**
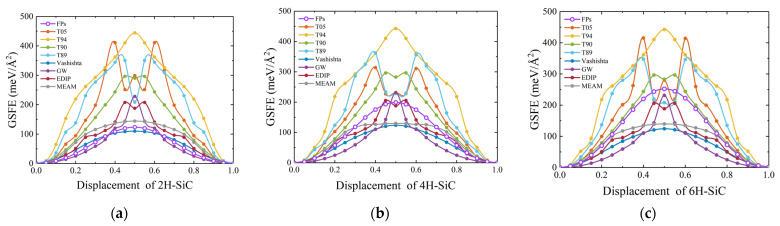
Generalized stacking fault energy for hexagonal SiC. (**a**), (**b**), and (**c**) show the variation of GSFE with displacement for 2H-, 4H-, and 6H-SiC, respectively.

In hexagonal SiC, when nanoindentation occurs on the (0001) basal plane, slip is observed along the $\left[11\bar{2}0\right]$ direction [79]. Thus, for hexagonal SiC, we primarily calculated the variation of GSFE with displacement for the slip system along the *a*-axis ($\left[11\bar{2}0\right]$ direction) during rigid body movement. The DFT results indicate that the GSFE for 2H-, 4H-, and 6H-SiC gradually increases, as shown in Figure 20. The stacking fault energy of 4H-SiC calculated using the Vashishta potential is in close agreement with the calculations reported by previous researchers [24]. The results obtained for the three phases using empirical potentials exhibit remarkable similarity. Overall, the Vashishta potential is consistent in the shape of GSFE curves. The structure of the dislocation core is closely associated with the shape of GSFE [27,40]. Therefore, the Vashishta potential is often used for the nanoindentation simulation of hexagonal SiC. The GSFE is closely related to the mechanism of dislocation motion in materials. For instance, when calculated using the T89 potential, the GSFE results for 4H-SiC and 6H-SiC are highly similar. Consequently, under the interactions of this interatomic potential, the stress–strain curves obtained from nanoindentation simulations for 4H-SiC and 6H-SiC would exhibit a close resemblance [79].

## 4. Conclusions

To assess the ability of multiple interatomic potentials to describe the properties of four common SiC structures, elastic constants, moduli, vibrational properties, thermal conductivities, point defect formation energies, point defect migration energies, surface energies, and GSFE were calculated using MD methods. The results show that the T05 potential agrees with both the DFT and experimental results in terms of elastic constant, modulus, brittle/toughness, and Vickers hardness, which implies that this interatomic potential is more suitable for mechanical simulations of SiC. The better prediction of the elastic constants allows the T05 potential to obtain more accurate Debye temperatures. The Vashishta, EDIP, and MEAM potentials effectively describe the lattice vibrational properties of SiC. At the macroscopic level, the thermal conductivity of SiC obtained from the T90 potential agrees well with the FPs and the experimental results. In terms of point defects of 3C-SiC, all interatomic potentials can only describe the formation energies of some point defects. The EDIP potential provides more accurate point defect formation energies in hexagonal SiC. For point defect migration, the GW potential is more advantageous. Thus, the GW potential appears to be more suitable for the cascaded collision simulation of hexagonal SiC. The T90 and T94 potentials describe the surface energy of the three low-index surfaces of 3C-SiC well. The results of the GSFE with the Vashishta potential are consistent with the DFT, which indicates that this potential is also suitable for the mechanical simulation of the impact and polishing of hexagonal SiC. This study is helpful for selecting interatomic potentials in SiC simulation.

## Figures and Tables

**Table 1:** Lattice parameters *a* and *c* (Å) as well as cohesive energies *E*_c_ (eV) calculated using interatomic potentials. The experimental and DFT results are also listed.

Phases		Experiments	DFT [9]	T05	T94	T90	T89	Vashishta	GW	EDIP	MEAM
3C	*a*	4.358 [54]	4.380	4.359 [11]	4.280 [26]	4.307	4.321 [26]	4.3582 [55]	4.360 [11]	4.364 [16]	4.360 [17]
	*E*_c_	—	−6.493	−6.339 [11]	−6.434 [26]	−6.210	−6.165 [26]	−6.3401 [55]	−6.412 [11]	−6.338 [16]	−6.375
2H	*a*	3.079 [56]	3.092	3.0825 [11]	3.026	3.046	3.056	3.0647 [55]	3.083	3.085	3.055 [17]
	*c*	5.053 [56]	5.074	5.0337 [11]	4.9416 [55]	4.9734	4.9895	5.0046 [55]	5.035	5.037	5.122 [17]
	*E*_c_	—	−6.490	−6.3392 [11]	−6.434 [55]	−6.210	−6.165	−6.3209 [55]	−6.412	−6.338	−6.367
4H	*a*	3.080 [57]	3.094	3.059 [11]	3.026	3.046	3.032 [26]	3.074	3.083	3.085 [16]	3.062 [17]
	*c*	10.082 [57]	10.129	10.225 [11]	9.883	9.947	10.135 [26]	10.063	10.069	10.074 [16]	10.20 [17]
	*E*_c_	—	−6.494	−6.339	−6.434	−6.210	−6.165	−6.326	−6.412	−6.338 [16]	−6.372
6H	*a*	3.081 [58]	3.095	3.078 [11]	3.026	3.046	3.051 [26]	3.077	3.083	3.085 [16]	3.067 [17]
	*c*	15.125 [58]	15.186	15.145 [11]	14.825	14.920	15.012 [26]	15.096	15.103	15.112 [16]	15.246 [17]
	*E*_c_	—	−6.494	−6.339	−6.434	−6.210	−6.165	−6.331	−6.412	−6.338 [16]	−6.371

**Table 2:** Calculated elastic constants *C_ij_* (GPa), bulk modulus *B* (GPa), shear modulus *G* (GPa), Young’s modulus *Y* (GPa), Poisson’s ratio *ν*, the rate of *B*/*G*, and Vickers hardness *H*_V_ (GPa) for 3C-SiC. The results of the experiment and DFT calculation are listed as well.

	Experiments [60]	DFT [9]	T05	T94	T90	T89	Vashishta	GW	EDIP	MEAM
*C*_11_	390	383.4	382 [11]	446.8 [12]	426.0 [13]	436.8 [14]	390 [15]	254 [11]	394 [16]	397 [17]
*C*_12_	142	126.8	145 [11]	138.2 [12]	133.8 [13]	118.0 [14]	142.6 [15]	225 [11]	142 [16]	147 [17]
*C*_44_	256	240.4	240 [11]	292.8 [12]	280.3 [13]	310.9 [14]	191.0 [15]	66 [11]	168 [16]	136 [17]
*B*	225	212.3	224 [11]	241.1 [12]	231.2 [13]	224.3 [14]	225.2 [15]	235 [11]	224 [16]	230 [17]
*G*	124	186	180.80	226.42	215.80	237.78	160.47	36.33	149.72	131.49
*Y*	448	433.4	427.40	517.31	493.78	527.07	388.97	103.65	367.91	331.40
ν	0.267	0.16	0.18	0.14	0.14	0.11	0.21	0.43	0.23	0.26
*B*/*G*	—	1.14	1.24	1.06	1.07	0.94	1.40	6.46	1.51	1.75
*H*_V_	26 ± 2 [59]	28.2	29.56	41.34	39.80	49.58	23.26	−1.16	20.14	15.02

**Table 3:** Calculated elastic constants *C_ij_* (GPa), bulk modulus *B* (GPa), shear modulus *G* (GPa), Young’s modulus *Y* (GPa), Poisson’s ratio *ν*, the rate of *B*/*G*, and Vickers hardness *H*_V_ (GPa) for 2H-SiC. The results of the experiment and theoretical calculation are listed as well.

	DFT [9]	T05	T94	T90	T89	Vashishta	GW	EDIP	MEAM
*C*_11_	492.1	483 [11]	506.09	482.92	507 [14]	415.24	324.37	436.80	472 [17]
*C*_12_	101.5	124 [11]	122.58	118.82	97 [14]	158.08	212.05	128.60	220 [17]
*C*_13_	49.5	67 [11]	94.25	91.52	42 [14]	150.94	167.13	112.23	2 [17]
*C*_33_	531.8	540 [11]	534.42	510.23	532 [14]	376.46	369.30	453.17	674 [17]
*C*_44_	151.0	164 [11]	176.19	167.14	196 [14]	126.65	49.06	142.33	104 [17]
*B*	213.0	224.67	240.97	231.09	211.93	236.00	234.52	225.88	230 [17]
*G*	183.62	183.04	190.57	180.93	209.53	126.10	60.13	152.33	145.56
*Y*	428.9	431.85	452.43	430.46	472.78	321.11	166.18	373.11	360.48
ν	0.16	0.18	0.19	0.19	0.13	0.27	0.38	0.22	0.24
*B*/*G*	1.16	1.23	1.26	1.28	1.01	1.87	3.90	1.48	1.58
*H*_V_	27.8	30.15	29.78	28.43	41.99	13.27	1.47	20.87	18.62

**Table 4:** Calculated elastic constants *C_ij_* (GPa), bulk modulus *B* (GPa), shear modulus *G* (GPa), Young’s modulus *Y* (GPa), Poisson’s ratio *ν*, the rate of *B*/*G*, and Vickers hardness *H*_V_ (GPa) for 4H-SiC. The results of the experiment and theoretical calculation are listed as well.

	Experiments [61]	DFT [9]	T05	T94	T90	T89	Vashishta	GW	EDIP	MEAM
*C*_11_	501	486.6	486 [11]	506.54	483.35	520 [14]	409.86	320.04	521 [16]	449 [17]
*C*_12_	111	103.4	127 [11]	122.12	118.39	94 [14]	145.11	216.39	105 [16]	193 [17]
*C*_13_	52	50.7	62 [11]	94.25	91.53	40 [14]	150.82	167.13	62 [16]	51 [17]
*C*_33_	553	531.7	538 [11]	534.42	510.23	533 [14]	455.48	369.30	567 [16]	576 [17]
*C*_44_	163	157.9	162 [11]	176.19	167.14	195 [14]	138.82	49.06	170 [16]	107 [17]
*B*	215	212.7	223.54	240.97	231.09	209.84	240.63	234.52	227 [16]	230 [17]
*G*	131.4	186.58	182.73	190.75	181.10	209.22	137.13	58.29	199.43	139.13
*Y*	347	431.8	430.80	452.77	430.78	471.09	345.71	161.50	463.98	347.18
ν	0.231	0.16	0.18	0.19	0.19	0.13	0.26	0.39	0.16	0.25
*B*/*G*	—	1.14	1.22	1.26	1.28	1.00	1.75	4.02	1.15	1.65
*H*_V_	—	28.1	30.25	29.83	28.48	42.40	15.43	1.23	34.55	17.00

**Table 5:** Calculated elastic constants *C_ij_* (GPa), bulk modulus *B* (GPa), shear modulus *G* (GPa), Young’s modulus *Y* (GPa), Poisson’s ratio *ν*, the rate of *B*/*G*, and Vickers hardness *H*_V_ (GPa) for 6H-SiC. The results of experiment and theoretical calculation are listed as well.

	Experiments [61]	DFT [9]	T05	T94	T90	T89	Vashishta	GW	EDIP	MEAM
*C*_11_	501	485.0	483 [11]	506.70	483.50	520 [14]	407.43	318.59	436.48	431 [17]
*C*_12_	111.5	104.1	125 [11]	121.97	118.25	97 [14]	142.73	217.84	128.92	173 [17]
*C*_13_	52	50.8	65 [11]	94.25	91.53	50 [14]	145.06	167.13	112.23	86 [17]
*C*_33_	553	533.0	546 [11]	534.42	510.23	561 [14]	439.08	369.30	453.17	513 [17]
*C*_44_	163	160.4	161 [11]	176.19	167.14	193 [14]	135.16	49.06	142.33	111 [17]
*B*	160.4	212.7	224.67	240.97	231.09	221.66	235.36	234.52	225.88	230 [17]
*G*	212.7	186.58	182.05	190.81	181.16	211.96	135.22	57.66	152.20	134.90
*Y*	186.58	433.2	430.00	452.89	430.88	482.18	340.45	159.88	372.86	338.38
ν	433.2	0.16	0.18	0.19	0.19	0.14	0.26	0.39	0.22	0.25
*B*/*G*	—	1.14	1.23	1.26	1.28	1.05	1.74	4.07	1.48	1.70
*H*_V_	—	28.2	29.84	29.85	28.50	40.56	15.45	1.15	20.83	15.93

**Table 6:** Transverse wave velocity *v_t_* (m/s), longitudinal wave velocity *v_l_* (m/s), average wave velocity *v_m_* (m/s), and Debye temperature *θ_D_* (K) calculated with different interatomic potentials for 3C-, 2H-, 4H-, and 6H-SiC.

Phases		DFT [9]	T05	T94	T90	T89	Vashishta	GW	EDIP	MEAM
3C	*v_t_*	7670	7497.9	8161.6	8044.9	8485.7	7061.0	3361.9	6830.2	6394.5
	*v_l_*	12,060	12,025.4	12,638.7	12,475.3	12,803.2	11,679.3	9384.7	11,516.3	11,231.5
	*v_m_*	8440	8261.8	8957.5	8830.9	9283.4	7805.4	3819.4	7564.3	7107.6
	*θ_D_*	1146.8	1128.8	1246.6	1221.2	1279.6	1066.7	521.8	1032.8	971.1
2H	*v_t_*	7620	7544.8	7650.9	7460.4	8033.1	6206.5	4324.8	6889.6	6725.6
	*v_l_*	12,020	12,073.4	12,331.6	12,053.9	12,300.9	11,110.8	9893.8	11,561.6	11,473.6
	*v_m_*	8380	8311.4	8434.9	8227.0	8804.6	6909.5	4883.6	7626.6	7456.5
	*θ_D_*	1146.8	1135.5	1157.1	1128.1	1206.8	949.6	667.1	1041.3	1019.0
4H	*v_t_*	7650	7537.8	7645.5	7453.1	7959.8	6511.4	4258.3	7883.0	6575.9
	*v_l_*	12,050	12,052.6	12,320.1	12,039.3	12,166.5	11,442.5	9855.4	12,426.7	11,354.7
	*v_m_*	8410	8303.0	8428.8	8218.7	8722.2	7237.9	4810.8	8669.6	7298.6
	*θ_D_*	1152.4	1134.4	1157.2	1128.0	1202.2	990.8	657.2	1183.7	997.3
6H	*v_t_*	7670	7523.8	7644.5	7451.1	8011.6	6471.0	4235.3	6886.7	6475.8
	*v_l_*	12,060	12,055.5	12,317.5	12,035.3	12,357.5	11,345.5	9842.4	11,559.3	11,280.0
	*v_m_*	8430	8289.5	8427.6	8216.5	8788.7	7191.6	4785.5	7623.6	7192.9
	*θ_D_*	1155.3	1132.6	1157.3	1128.0	1211.4	983.9	653.7	1040.9	982.8

**Table 7:** Phonon frequencies ν (THz) in high-symmetry points of the Brillouin zone calculated using different potentials and results of DFT and experiments for 3C-SiC.

	Experiments	DFT	T05	T94	T90	T89	Vashishta	GW	EDIP	MEAM
ν_TO_ (*Γ*)	23.88 [62]	23.01	28.70	32.11	31.44	28.39	26.14	26.06	31.90	33.17
ν_LO_ (*Γ*)	29.13 [63]	28.06	28.90	32.37	31.85	28.58	27.28	26.44	32.15	33.37
ν_TA_ (*X*)	11.18 [62]	10.93	13.71	14.40	14.15	14.34	11.90	8.20	12.59	10.12
ν_LA_ (*X*)	19.19 [62]	18.34	17.68	18.81	18.34	18.78	18.82	15.86	18.34	18.26
ν_TA_ (*K*)	13.50 [62]	13.65	15.71	16.65	16.13	15.79	15.47	9.32	14.81	12.49
ν_LA_ (*K*)	16.95 [62]	16.65	16.65	17.99	17.67	17.69	17.22	14.71	16.79	16.24
ν_TA_ (*L*)	7.95 [62]	7.99	9.44	10.12	9.86	10.27	7.73	5.00	9.13	8.30
ν_LA_ (*L*)	18.45 [62]	17.82	17.90	18.87	18.39	18.36	18.70	14.48	17.62	15.78

**Table 8:** Formation energy (eV) of different point defects for 3C-SiC, calculated by DFT and empirical potentials. The “—” represents an unstable structure.

Defects	DFT	T05 [11]	T94	T90	T89 [11]	GW	EDIP [16]	MEAM
V_C_	4.70 [70], 3.63 [71], 4.23 [52]	1.90	4.52	3.99	3.88	3.45	1.45	1.44 [17]
V_Si_	8.12 [70], 7.48 [71], 8.40 [52]	4.55	8.24	7.79	3.29	6.89	4.18	4.52 [17]
C_Si_	3.30 [70], 3.48 [71], 4.16 [52]	2.42	2.53	2.06	2.20	4.58	2.40	2.02 [17]
Si_C_	4.30 [70], 4.02 [71], 3.37 [52]	2.48	5.84	3.22	4.50	5.02	2.74	4.63 [17]
CT_C_	10.98 [70], 11.16 [52]	12.63	7.17	13.40	7.21	3.95	—	2.67 [17]
CT_Si_	10.09 [70], 10.32 [52]	9.38	4.13	16.35	4.40	3.61	6.40	8.74 [17]
SiT_C_	9.01 [70], 7.04 [71], 8.21 [52]	17.55	17.17	14.80	17.67	0.36	—	3.61 [17]
SiT_Si_	10.87 [70], 9.23 [71], 10.04 [52]	17.30	17.71	16.69	15.89	3.16	—	4.38 [17]
C-C + <100>	7.06 [70], 6.31 [71], 7.33 [52]	4.78	9.85	7.99	6.50	2.35	4.82	7.72
C-C + <110>	7.19 [70], 6.65 [71], 7.51 [52]	10.28	8.29	12.61	5.68	2.60	5.13	4.07
Si-C + <100>	7.62 [70], 6.94 [71]	8.31	11.82	12.15	7.69	2.73	4.67	—
Si-Si + <100>	10.36 [70], 9.32 [71], 9.67 [52]	20.90	16.65	9.50	12.52	1.89	8.25	8.28
Si-Si + <110>	9.07 [70],8.11 [71], 8.35 [52]	17.73	17.12	15.02	12.11	1.47	—	10.92
C-Si + <100>	11.08 [70]	—	—	10.91	—	1.23	8.90	4.26
C-Si + <110>	9.42 [70]	—	—	15.48	12.39	−0.23	7.78	—

## Data Availability

The data that support the findings of this study are available from the corresponding author upon reasonable request.

## Supplementary Materials

The following supporting information can be downloaded at: https://www.mdpi.com/article/10.3390/ma17010150/s1, Table S1: Point defect formation energy (eV) of 2H-SiC; Table S2: Point defect formation energy (eV) of 4H-SiC; Table S3: Point defect formation energy (eV) of 6H-SiC; Input script for LAMMPS.
