# Supplementary material for: Comparison and Assessment of Different Interatomic Potentials for Simulation of Silicon Carbide
Source: Materials (Basel). 2023 Dec 27;17(1):150. doi: 10.3390/ma17010150 (PMC10779864; doi:10.3390/ma17010150)
Supplement: Supplementary file 1 [file materials-17-00150-s001.zip › Table of contents.pdf]

Table of contents

Table S1. Point defect formation energy (eV) of 2H-SiC. P1
Table S2. Point defect formation energy (eV) of 4H-SiC. P1
Table S3. Point defect formation energy (eV) of 6H-SiC. P1
The LAMMPS input script is in the compressed package named input script.

Table S1. Point defect formation energy (eV) of 2H-SiC.

	DFT	T05	T94	T90	T89	GW	EDIP	MEAM
h_V_{C}	4.81	2.23	4.51	3.99	4.05	3.46	1.88	1.71
h_V_{Si}	7.52	4.89	8.24	7.79	3.31	6.90	4.60	4.87
h_Si_{C}	4.25	2.14	5.74	3.18	4.14	4.90	2.71	3.91
h_C_{Si}	3.54	2.18	2.52	2.06	2.19	4.60	2.38	2.01
h_I_{C}	6.44	4.89	9.85	17.21	6.20	2.36	4.53	2.95
h_I_{Si}	10.15	16.25	16.12	14.00	15.89	1.17	13.25	2.84

Table S2. Point defect formation energy (eV) of 4H-SiC.

	DFT[1]	T05	T94	T90	T89	GW	EDIP	MEAM
h_V_{C}	4.88	2.24	4.52	4.00	4.04	3.45	1.88	1.55
h_V_{Si}	7.1	4.87	8.24	7.79	3.29	6.89	4.60	4.87
h_Si_{C}	4.63	2.27	5.90	3.22	4.35	4.88	2.72	4.59
h_C_{Si}	2.65	2.17	2.53	2.06	2.22	4.59	2.38	1.80
h_I_{C}	6.21	4.77	10.05	18.70	6.08	2.34	4.48	2.84
h_I_{Si}	10.87	16.08	16.18	13.98	15.76	1.17	13.23	2.73
k_V_{C}	4.84	2.25	4.51	3.99	4.04	3.46	1.88	1.78
k_V_{Si}	7.16	4.87	8.24	7.79	3.29	6.90	4.60	4.83
k_Si_{C}	4.58	2.25	5.80	3.19	4.29	4.96	2.71	3.45
k_C_{Si}	2.61	2.19	2.53	2.06	2.22	4.60	2.39	1.95
k_I_{C}	6.32	10.32	7.91	14.63	7.86	2.68	4.84	3.73
k_I_{Si}	12.46	12.72	18.27	15.89	13.76	1.46	11.17	3.16

Table S3. Point defect formation energy (eV) of 6H-SiC.

	DFT	T05	T94	T90	T89	GW	EDIP	MEAM
h_V_{C}	4.73	2.24	4.52	4.00	4.05	3.45	1.88	1.69
h_V_{Si}	7.52	4.89	8.24	7.79	3.30	6.89	4.60	4.89
h_Si_{C}	4.19	2.25	5.83	3.22	4.27	4.87	2.72	4.54
h_C_{Si}	3.55	2.17	2.53	2.06	2.19	4.59	2.38	1.98
h_I_{C}	6.57	4.75	9.89	18.51	5.96	2.34	4.48	2.78
h_I_{Si}	9.02	16.15	16.33	13.98	15.76	1.16	13.21	2.67
k_V_{C}	4.62	2.24	4.51	3.99	4.05	3.45	1.88	1.82
k_V_{Si}	7.52	4.89	8.24	7.79	3.30	6.89	4.60	4.89
k_Si_{C}	4.17	2.16	5.68	3.18	4.17	4.89	2.71	4.52
k_C_{Si}	3.54	2.18	2.53	2.06	2.20	4.59	2.38	1.97

k_I_C	6.56	10.45	5.86	14.61	7.98	2.71	4.79	3.68
k_I_{Si}	9.92	17.13	17.57	14.82	17.48	0.36	12.56	3.29

[1] Kobayashi T, Harada K, Kumagai Y, et al. Native point defects and carbon clusters in 4H-SiC: A hybrid functional study [J]. J Appl Phys, 2019, 125(12): 125701.
